# Social and Non-social Reward Processing and Depressive Symptoms Among Sexual Minority Adolescents

**DOI:** 10.3389/fnbeh.2019.00209

**Published:** 2019-09-13

**Authors:** Kristen L. Eckstrand, Luis E. Flores Jr., Marissa Cross, Jennifer S. Silk, Nicholas B. Allen, Kati L. Healey, Michael P. Marshal, Erika E. Forbes

**Affiliations:** ^1^Department of Psychiatry, University of Pittsburgh, Pittsburgh, PA, United States; ^2^Department of Psychology, Queen’s University, Kingston, ON, Canada; ^3^Department of Psychology, University of Pittsburgh, Pittsburgh, PA, United States; ^4^Department of Psychology, University of Oregon, Eugene, OR, United States; ^5^Department of Psychiatry and Behavioral Sciences, Duke University, Durham, NC, United States

**Keywords:** depression, adolescence, social reward, LGBT, fMRI

## Abstract

Sexual minority adolescents (SMA) are more likely to suffer from depression, putatively through experiences of social stress and victimization interfering with processing of social reward. Alterations in neural reward networks, which develop during adolescence, confer risk for the development of depression. Employing both social and monetary reward fMRI tasks, this is the first neuroimaging study to examine function in reward circuitry as a potential mechanism of mental health disparities between SMA and heterosexual adolescents. Eight SMA and 38 heterosexual typically developing adolescents completed self-report measures of depression and victimization, and underwent fMRI during monetary and peer social reward tasks in which they received positive monetary or social feedback, respectively. Compared with heterosexual adolescents, SMA had greater interpersonal depressive symptoms and exhibited blunted neural responses to social, but not monetary, reward in socioaffective processing regions that are associated with depressive symptoms. Specifically, compared with heterosexual adolescents, SMA exhibited decreased activation in the right medial prefrontal cortex, left anterior insula (AI), and right temporoparietal junction (TPJ) in response to being liked. Lower response in the right TPJ was associated with greater interpersonal depressive symptoms. These results suggest that interpersonal difficulties and the underlying substrates of response to social reward (perhaps more so than response to monetary reward) may confer risk for development of depressive symptoms in SMA.

## Introduction

Sexual minority adolescents (SMA), including those who identify as lesbian, gay, or bisexual, are four times more likely to meet criteria for Major Depressive Disorder and are at three times greater risk for suicidal thoughts and behaviors compared with their heterosexual peers (Fergusson et al., 1999; Marshal et al., 2011; Burton et al., 2013). Minority stress theory posits that mental health concerns among SMA arise in part as a response to interpersonal stress and victimization (Meyer, 2003). SMA are more likely to experience interpersonal stress and victimization compared with heterosexual peers, and victimization is correlated with greater depression and suicidal ideation that persist into young adulthood (Burton et al., 2013). Expectations of interpersonal rejection are further associated with depression (Feinstein et al., 2012). These adverse experiences emerge at a younger age in SMA than heterosexual peers, sometimes even prior to adolescence.

Depression among SMA is not simply the result of negative interpersonal interactions, but also is postulated to arise from the paucity of positive interactions and social support. SMA who do not have openly accepting and supportive families experience greater depressive symptoms and suicidal ideation than those who do (Ryan et al., 2010). SMA who live in less socially supportive environments are more likely to attempt suicide than those living in supportive environments (Williams et al., 2005; Hatzenbuehler, 2011). Even after disclosing their sexual orientation to others (“coming out”), SMA who perceive that they are not accepted or are a “burden” to others experience greater depressive symptoms and suicidal ideation (Baams et al., 2015). Altogether, these findings suggest that depression among LGB youth results from both the presence of interpersonal stress and disruption of reward, particularly during social situations (e.g., receiving social approval).

Typically developing adolescents tend to exhibit heightened reward function compared with children and adults (Somerville et al., 2010) due to the asynchronous development of “traditional” neural reward circuits [including e.g., ventral striatum, dorsomedial prefrontal cortex (dmPFC)] and self-regulation circuits [including e.g., dorsolateral and ventrolateral prefrontal cortex (vlPFC); Forbes and Dahl, 2012]. As adolescents gain independence, develop greater social orientation, and desire social status gains, these frontostriatal reward networks are particularly influenced by social reward neurocircuitry [e.g., temporoparietal junction (TPJ), anterior insula (AI); Blakemore, 2008]. While social development plays a critical role in typical adolescent brain development, social experiences including victimization, prosocial behavior, low parental support, peer liking and rejection, and social stress are associated with altered activity in both neural reward and social circuits (Auerbach et al., 2014; Casement et al., 2014; Morelli et al., 2014; Telzer et al., 2014). Further, changes in neural reward and social circuits have been associated with the development of depressive symptoms in adolescence (Forbes et al., 2009; Healey et al., 2014; Miller et al., 2015; Stringaris et al., 2015). Together, these literatures suggest that adolescents’ altered neural response to reward in general may confer risk for the development of depressive symptoms, and this risk may be influenced by particularly salient social experiences—such as being liked by peers.

The social context of reward could be especially salient for SMA given their unique experiences of social stress and the importance of acceptance with parents, peers, and even romantic/sexual partners. Despite the fact that SMA have unique social experiences including victimization, are more likely to experience depressive symptoms compared with heterosexual peers, and that sexual orientation is typically self-identified and communicated to others beginning in adolescence, the neural circuitry underlying the interplay between social acceptance and the development of depression has not yet been investigated. In this first neuroimaging study comparing SMA and heterosexual youth, we explored neural response to differing reward contexts (e.g., monetary and social reward), victimization, and depressive symptoms between SMA and heterosexual adolescents. We specifically hypothesized that SMA would exhibit decreased activation in social reward neural circuits in response to being liked by peers but would not exhibit these differing responses to monetary reward. We further hypothesized that these altered patterns would be associated with self-reported depressive symptoms. Lastly, we hypothesized that victimization would moderate the association between altered neural activation and self-reported depressive symptoms, where individuals who have experienced victimization would demonstrate the relationship between altered neural activation and self-reported depressive symptoms.

## Materials and Methods

### Participants

Seventy adolescents aged 14–18 with no history of psychiatric disorder/treatment or serious medical problems were recruited from community settings to participate in a study on social reward processing in typically developing adolescents. Of the recruited 70 participants, 46 completed all behavioral, self-report, and neuroimaging measures and were included in the final sample of the present study [19M, 27F (16.3 ± 1.4 years), 65% white/Caucasian, 24% black/African American, 11% mixed racial background]. Recruited individuals were excluded from the final sample (*n* = 24) if they did not complete the fMRI scan due to scanning exclusionary criteria (*n* = 8; three due to recent concussion, three due to claustrophobia, two due to mental health diagnosis), could not be contacted or withdrew from the study after their initial behavioral assessment (*n* = 5), did not complete the fMRI task or were removed due to scan quality (*n* = 8), or had missing behavioral data (*n* = 3). Male and female participants did not differ in age or race. The University of Pittsburgh IRB approved all research procedures and written informed consent was obtained from each participant and a parent or guardian.

### Measures

#### Sexual Orientation Identity

All participants answered the single question, “What is your sexual identity?” with one of the following: 100% Heterosexual (Straight), Mostly Heterosexual (Straight, but somewhat attracted to people of your own sex), Bisexual (Attracted to men and women equally), Mostly Homosexual (Gay, but somewhat attracted to people of the opposite sex), 100% Homosexual (Gay or Lesbian). This demographic question is equivalent to that used in the National Longitudinal Study of Adolescent Health (Chen and Chantala, 2014), defining sexual orientation identity in terms of same-sex sexual attraction. This approach was used as prior data has demonstrated that adolescents’ endorsement of same-sex attraction and same-sex sexual identity varies across adolescent development (Marshal et al., 2013), and only assessing one measure may incorrectly identify SMA as heterosexual. All individuals who identified with a non-same sex identity or attraction were classified as SMA.

#### Depressive Symptoms

Participants completed the Center for Epidemiologic Studies Depression Scale (Radloff, 1977; CES-D) to assess depressive symptoms. The CES-D is a 20-item self-report scale, where higher scores indicate the presence of more symptomatology. Four previously determined factors of the CES-D were examined: depressed affect, positive affect, somatic symptoms, and interpersonal difficulty (Radloff, 1977).

#### Victimization

The Youth Risk Behavior Survey (YRBS) 2009 (Eaton et al., 2010) is a validated epidemiologic self-report instrument assessing health-risk behaviors in high school students, including victimization. All participants completed the YRBS, and victimization was calculated based on a previously identified YRBS measure items of victimization among SMA (Russell et al., 2014). Items assessing victimization were related to fighting, bullying, and safety (see Supplementary Material). The composite measure of victimization was calculated as the standardized mean of these individual items.

#### Social Reward Task

Participants completed an fMRI social reward task to investigate neural response to positive social feedback as previously described in Healey et al. (2014). Prior to scanning, participants rated photos of other adolescents (40 photos; 50% female) based on how much they thought they would like the individuals in the photos (1 = “not at all” to 9 = “very much”); participants were told their photos would be “rated” by the other adolescents. A personalized stimulus set was created for each participant containing blocks of *positive feedback*, where participants received feedback that their peers rated them favorably and *neutral feedback* where they were informed that peers had not yet rated them.

Personalized stimulus sets were presented in a block design composed of *positive feedback* and *neutral feedback* blocks. Each of the 32 stimuli was presented three times over eight blocks, with each block consisting of 12 stimuli and lasting 84 s. Of the eight blocks, four were *positive feedback* and four were *neutral feedback*. Each block also contained two images of the opposite stimulus type in order to minimize habituation and predictability from the block design (e.g., positive feedback blocks contained 10 *positive feedback* stimuli and two *neutral feedback* stimuli). Each image was presented for 3 s, with a jittered inter-trial interval between stimuli and an inter-block interval of 8 s. Participants were instructed to press a button every time they saw a face to confirm they were attending to the task. At the end of the scan, the deception of the task was disclosed, and participants were told that their image had not been rated by other adolescents.

#### Monetary Reward Task

Neural response to monetary reward was assessed using an adapted task card-guessing task (Delgado et al., 2000; Nusslock et al., 2012). In this event-related paradigm, each trial was comprised of an anticipation period and an outcome period, where potential outcomes could be a win, loss, or no-change trial. Participants were told that they would receive $1 for win trials, lose $0.50 in loss trials, and neither win nor lose money in the no-change trial. However, trials were fixed in a pseudorandomized fashion where all participants received the same number of win, loss, or no-change trials. Participants were unaware of the fixed outcomes.

Each trial began with a “decision” card containing a question mark symbol where participants had 4 s to guess, through button press, whether the value of a presented card was higher or lower than five. The anticipation phase began with a card presenting the trial type (reward or loss). After 6 s, the actual numerical value of the card (1–9) was presented (500 ms). The outcome phase was then shown (a green upward-facing arrow for win, a red downward-facing arrow for loss, or a yellow circle for neutral feedback; 500 ms) and a crosshair presented for 9 s. There were six trials of each outcome (i.e., win, loss, no win, no loss).

### fMRI Acquisition and Preprocessing

Participants were scanned using a Siemens 3T Trio scanner at the University of Pittsburgh Magnetic Resonance Research Center (MRRC). MPRAGE structural images were acquired with high-resolution T1-weighted images with 1 mm isometric voxels (TR/TE/flip angle = 2,300 ms/2.98 ms/9; FOV = 256 × 240; 1.2 mm slice; 160 slices; 256 × 240 matrix; 1 Nex). Functional blood oxygen level dependent (BOLD) images were acquired using gradient echo planar imaging (EPI) sequences: 39 oblique axial slices (3.1 mm thick, 0 mm gap) beginning at the cerebral vertex and encompassing the entire cerebrum and the majority of the cerebellum, oriented to the AC-PC line (TR/TE = 2,000 MS/30 ms, FOV = 205 × 205, matrix = 64 × 64). A reference EPI scan acquired prior to fMRI data collection was visually inspected for artifacts and signal quality.

Preprocessing and fMRI image analysis was performed using Statistical Parametric Mapping software, version 8^1^. Images for each subject were realigned, motion-corrected, and high-pass temporally filtered with a cut-off of 128 s Volumes with high motion and artifacts were adjusted using ART (volumes where average image intensity deviated >3SD from the mean intensity or where movement exceeded 0.5 mm in translation or 0.01° in rotation from the previous image^2^, Chai et al., 2014). The mean functional image was coregistered with the high-resolution 3D anatomic image, normalized to standard stereotactic space (Montreal Neurological Institute template) using a 12-parameter affine model, and spatially smoothed with a 6 mm full-width at half-maximum Gaussian filter.

### Data Analysis

#### Second Level fMRI

Neural response to being liked in the social reward task was determined for each individual by contrasting brain activity during receipt of positive feedback compared with blocks of neutral feedback (*positive > neutral feedback*), as this contrast reflects social reward and corresponds to adolescents’ experience of others’ evaluation in social settings (e.g., social media). Neural response to monetary reward was determined for each individual by contrasting brain activity during anticipation of a reward in a win condition compared with anticipation in neutral (no-change) conditions (*win anticipation > neutral*). Both of these contrasts have been demonstrated to reflect the neural reward response in social and monetary contexts, respectively, in adolescents (Nusslock et al., 2012; Healey et al., 2014). Whole-brain individual contrast images were entered into two separate two-sample *t*-tests to examine differences in brain activation between SMA and heterosexual adolescents in response to: (1) peer liking; and (2) monetary reward anticipation. Age, gender, and race were included as covariates in the model. Age was included as a continuous variable; gender (male, female) and race (white/Caucasian, black/African American, mixed racial background) were included as nominal variables. Given that socioaffective circuitry encompasses multiple neural regions across networks, whole brain analyses were used for second-level analyses.

Monte Carlo simulations using REST v1.8^3^ were used to estimate the minimum number of contiguous voxels per cluster (activated at *p*_unc_ < 0.005) corrected to avoid Type I error (*p*_corr_ < 0.05), resulting in a cluster extent threshold of 154 voxels for whole-brain analyses of social reward and 379 voxels for reward anticipation. As discussed in detail in the Supplementary Material, these thresholding criteria were selected because block designs with longer activity durations analyzed in SPM with two groups, as in the social reward task in this study, are less affected by cluster extent thresholding errors and are expected to yield a false positive rate of ≤5% (Eklund et al., 2016). Individual parameter estimates of the BOLD response for clusters reaching significance in second-level analyses were extracted using Marsbar^4^. One participant was removed from the analyses as an outlier, due to their BOLD signal cluster of interest >3 standard deviations from the mean.

#### Depressive Symptoms and Victimization

To address potential outliers, a 95% winsorization was applied to self-report scales where data points >3 standard deviations from the mean were replaced by values exactly 3 standard deviations from the mean. Across depressive symptom subscales and victimization, only one data point required winsorizing (see Supplementary Figure S1). Winsorizing this data point did not affect the significance of group differences (see Supplementary Table S2). As expected from a typically-developing sample, behavioral data were not normally distributed using the Kolmogorov–Smirnov test and were instead skewed towards having lower or no symptoms (*CES-D Subscales*: somatic symptoms, *D*_(46)_ = 0.154, *p* = 0.008; depressed affect, *D*_(46)_ = 0.252, *p* < 0.001; positive affect, *D*_(46)_ = 0.175, *p* = 0.001; interpersonal difficulty, *D*_(46)_ = 0.402, *p* < 0.001; *Victimization*: *D*_(46)_ = 0.449, *p* < 0.001). As such, differences in depressive symptoms based on sexual orientation and victimization were determined using non-parametric two-sample tests with bootstrapping (*n* = 10,000 resamples) implemented in SPSS v23. As behavioral variables were not normally distributed, a multiple linear regression analysis with bootstrapping (*n* = 10,000 resamples) was performed to test the robustness of the association between neural activation in areas where activation differed by sexual orientation and depressive symptoms (Fox, 2002). The four CES-D depressive symptom subscales were included as dependent variables and neural activation in the aforementioned regions were included as independent variables in the single model. Because neural activation measures had already been corrected for demographic variables, these variables were not re-entered into the multiple linear regression model. Finally, the PROCESS macro (Hayes, 2013) was used to test the moderating effect of victimization.

## Results

### No Difference in Demographic Factors Between SMA and Heterosexual Adolescents

Of the 46 adolescents included in the final sample, 38 identified with the sexual identity of “100% Heterosexual” whereas the remaining 8 identified with a sexual minority sexual identity. There was no difference between heterosexual adolescents and SMA by age (Heterosexual: 16.3 ± 1.4 years; SMA: 16.3 ± 1.2 years; *t*_(1,42)_ = 0.20, *p* = 0.84), gender (Heterosexual: 18M/20F; SMA: 1M/7F; (*χ*^2^ = 3.26, *p* = 0.07) or race (Heterosexual: 68% White, 24% Black/AA, 8% Multiracial; SMA: 50% White, 25% Black/AA, 25% Multiracial; *χ*^2^ = 2.17, *p* = 0.34).

### Greater Interpersonal Depressive Symptoms Among SMA

SMA had significantly greater interpersonal depressive symptoms compared with heterosexual adolescents, although did not differ on other CES-D depressive subscales (see Table 1). Depressive symptoms did not differ based by age, gender, or race. Ten participants experienced one or more instances of victimization (see Supplementary Table S1), including two SMA and eight heterosexual adolescents. Across participants, experiencing victimization was positively associated with interpersonal depressive symptoms (*R*^2^ = 0.08, *p* = 0.05) but not with other depressive symptoms. SMA did not experience greater victimization than heterosexual adolescents (*Z*_(1,45)_ = −0.48, *p* = 0.69). Males, however, did report more experiences of victimization compared with females (M: 0.21 ± 0.34, F: 0.04 ± 0.16; *Z*_(1,45)_ = −2.17, *p* = 0.03). Victimization was unrelated to age or race (see Table 1).

**Table 1:** Depressive symptoms and victimization by sexual orientation.

		Orientation^a^		Race^b^		Gender^a^		Age^c^	
		*Z*	*p*	*χ*^2^	*p*	*Z*	*p*	*F*	*p*
CES-D^†^	Somatic symptoms	−1.04	0.31	2.65	0.27	−0.11	0.91	0.36	0.55
	Depressive affect	−1.19	0.25	1.34	0.52	−0.49	0.63	0.45	0.50
	Positive affect	−0.28	0.79	0.30	0.99	−0.05	0.97	0.88	0.36
	Interpersonal difficulty	−2.18	**0.02**	0.66	0.73	−0.52	0.59	0.40	0.53
	Victimization^†^	−0.48	0.69	1.62	0.49	−2.17	**0.03**	1.50	0.23

*CES-D, Center for Epidemiologic Studies Depression Scale; SMA, sexual minority adolescents. ^†^Performed with bootstrapping (n = 10,000 resamples). ^a^Non-parametric two-sample test. ^b^Non-parametric k-sample test. ^c^Multiple linear regression. Bold values indicate p < 0.05*.

### SMA and Neural Response to Social and Monetary Reward

**Figure 1:**
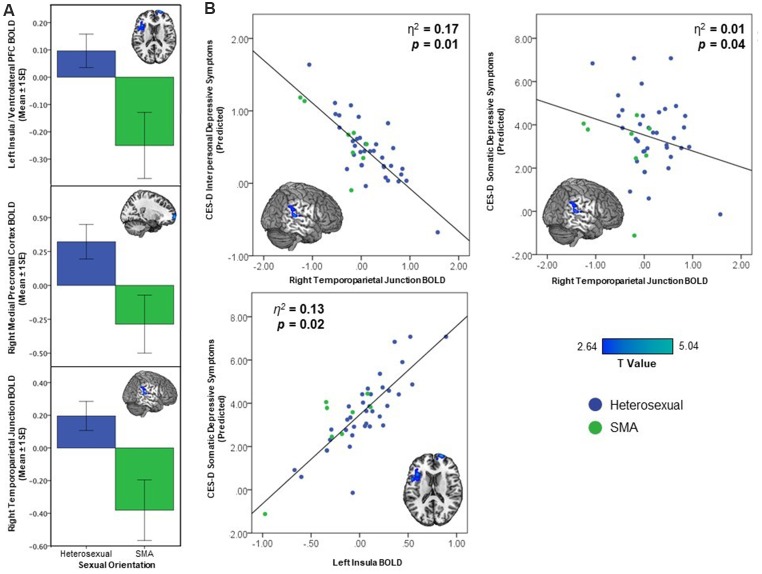
Activation differences between sexual minority adolescents (SMA) and heterosexual adolescents In response to being liked demonstrating **(A)** less activation in the right mPFC, left anterior insula (AI)/ventrolateral prefrontal cortex (vlPFC), and right temporoparietal junction (TPJ), and **(B)** greater depressive symptoms associated with decreasing brain activation in the right TPJ and increased depressive symptoms in left AI/vlPFC.

Whole brain analyses revealed differences based on sexual minority status in response to being liked (see Figure 1A). SMA exhibited less activation compared to their heterosexual peers in the right mPFC, left AI, and right TPJ during receipt of social reward (see Table 2). In contrast, there were no differences observed in neural activation to monetary reward anticipation between SMA and heterosexual adolescents. To test whether differences in reward response were isolated to social stimuli, we further examined neural response to monetary reward receipt (*win outcome > neutral outcome*) and did not observe differences between SMA and heterosexual adolescents.

**Table 2:** Activation differences in response to being liked between heterosexual and sexual minority adolescents (SMA), and relationship of BOLD response to depressive symptoms (CES-D).

Brain region	Hemi.	Vox.	Max T	x	y	z	Reg.	CES-D^†^				
								Somatic symptoms	Depressed affect	Positive affect	Interpersonal difficulty	*f*^2^
*Less activation in SMA* medial prefrontal cortex	R	360	5.05	18	66	−2	*β*	0.50	0.88	0.27	<−0.01	0.04
							*p*	0.47	0.25	0.70	0.99
Insula	L	456	4.38	−48	12	8	*β*	4.63	0.88	0.36	0.92	0.21
							*p*	**0.01**	0.60	0.88	0.07
Temporoparietal junction	R	160	3.81	62	−32	10	*β*	−1.95	−1.07	0.36	−0.82	0.27
							*p*	**0.02**	0.19	0.64	**0.05**	

*CES-D, Center for Epidemiologic Studies Depression Scale. ^†^Multiple linear regression performed using bootstrapping (n = 10,000 resamples). Bold values indicate p < 0.05*.

### Depression Association With Regions Distinguishing SMA and Heterosexual Adolescents

The three clusters that distinguished neural response to social reward in SMA and heterosexual adolescents were included in a multiple linear regression predicting depression subscales. The right TPJ—within the cluster whose activity distinguished SMA from heterosexual adolescents—was associated with depressive symptoms. Specifically, lower right TPJ activation was associated with higher interpersonal [*β* = −0.82, *p* = 0.05, 95% CI (−1.69, −0.18)] and somatic [*β* = −1.95, *p* = 0.02, 95% CI (−3.99, −0.55)] depressive symptoms (see Figure 1B, Table 2). Higher left insula activation was associated with greater somatic depressive symptoms [*β* = 4.63, *p* = 0.01, 95% CI (1.52, 8.35)]. While demographic variables were not re-included as covariates in the multiple linear regression model given neural activation values were already corrected for these variables, their inclusion in the multiple linear regression model did not change the significance of the above findings (see Supplementary Table S3). Contrary to our hypothesis, victimization did not moderate these associations (*F*_(1,41)_ = 1.15, *p* = 0.29).

## Discussion

This is the first study to examine differences in brain function between sexual minority and heterosexual typically developing adolescents. The findings from this study suggest that SMA—who experienced greater interpersonal depressive symptoms compared with heterosexual adolescents—may exhibit altered function in salience and social processing networks in response to social reward compared with heterosexual adolescents. Furthermore, blunted neural response in the right TPJ—a region implicated in perspective-taking and processing social information—was associated with higher interpersonal depressive symptom severity.

SMA are a population at high risk for depression, and they are more likely than heterosexual youth to have experienced social stressors, such as interpersonal victimization and rejection (Burton et al., 2013). Consistent with this, sexual minority status in our sample remained the key predictor of interpersonal depressive symptoms whereas age, gender, and race did not.

The present study provides preliminary support for neural correlates of these disparities, demonstrating decreased activation to social reward in mPFC, AI, vlPFC, and TPJ in a sample of SMA. These regions are consistent with altered function in social reward circuitry in youth with depression (Forbes et al., 2009; Healey et al., 2014). Further, SMA experienced greater interpersonal depressive symptoms, and lower right TPJ response to social reward was associated with greater interpersonal depressive symptoms. In contrast, SMA did not exhibit differential patterns of the neural response to monetary reward anticipation. Decreased activation to monetary reward is also associated with depression in adolescence (Keren et al., 2018). This suggests that social reward processes may be particularly important in understanding the development of depression in SMA. Given that the current fMRI task involved peer feedback, participants were provided an opportunity for spontaneous perspective-taking including considering others’ perceptions of them. The right TPJ is critical for perspective-taking (Krall et al., 2015), and lower activation in this region in response to social reward suggests that SMA may be less engaged during socially rewarding feedback compared with heterosexual youth. Such cognitive disengagement in rewarding social situations may explain depression-associated lower TPJ response in SMA and subsequently the heightened interpersonal depressive symptoms experienced by SMA.

It is important to note that while deactivation in the right TPJ—a region in which SMA demonstrated decreased activation compared to heterosexual adolescents—was associated with greater depressive symptom severity, deactivation in the AI was associated with *decreased* somatic symptom severity. This may be due to the differences in function between the TPJ and AI/vlPFC. Whereas the TPJ is a critical region in socioaffective processing, the AI is implicated in somatosensory, interoceptive, and salience processing (Smith et al., 2014). The decreased activation of the AI may be due to the prior experiences or expectation of peer rejection and subsequently decreased salience by SMA in response to being liked (Rudolph et al., 2016). However, greater activation of AI is expected with somatosensory experiences, including those associated with depressive symptoms (e.g., sleep, appetite). Alternatively, this finding raises the possibility that the specific activation patterns seen among SMA may confer both risk and resilience in the development of depression. Altogether, these findings indicate that SMA demonstrate altered activity in a network of regions with putative socioaffective function. Furthermore, this different pattern of neural activity might serve as a mechanism for increased risk for the social and affect regulation difficulties that are considered central to depression (Davey et al., 2008; Burnett et al., 2011; Auerbach et al., 2014). Future work is necessary to examine the role of SMA-related discrimination in socioaffective circuitry and, subsequently, affective states.

Unexpectedly, heterosexual adolescents and SMA did not differ in victimization experiences, and victimization did not moderate SMA effects on depression. This may be due to the measures of victimization included in the YRBS, which focus primarily on violent victimization. While this is one component of victimization is experienced by SMA, more specific scales have been developed to measure additional aspects of sexual minority-related stress (Newcomb and Mustanski, 2010; Goldbach et al., 2017). These scales, which include questions such as “There are times when I do not want to be LGBTQ” and “I expect people to reject me when they find out that I am LGBTQ,” measure a wider variety of victimization and negative interpersonal experiences not present in the YRBS (Goldbach et al., 2017). Further studies examining the impact of orientation-related stress on social reward circuits and depressive symptoms are necessary. Further, the focus on violent victimization may explain the observed effect of gender, as adolescent males typically have more experiences of violent victimization than females (Tillyer and Tillyer, 2016).

It is worth noting that few research studies examine multiple classes of reward in the same study, with the current results suggesting that depressive symptoms in SMA may be more related to social, but not monetary reward. However, there are clear differences between the two reward fMRI tasks utilized in this study that limit their direct comparison (e.g., the social reward task was a block design while the monetary reward task was an event-related design) and future studies may want to examine neural correlates of classes of rewarding stimuli using similarly designed fMRI tasks. Further, while the results do demonstrate differences in neural activation between SMA and heterosexual adolescents, the association between these regions and depressive symptoms does not specifically demonstrate an SMA-related pattern of depressive symptoms as the associations between activation and depressive symptoms were across the entire sample. While mPFC involvement in adolescent depression is well-recognized, the present study demonstrates mPFC deactivation among SMA in a more anteriorly located mPFC region (Etkin et al., 2011); it is currently unclear what role deactivation of this region has on social reward processing and depression. Again, additional work is necessary to understand how the unique social and environmental experiences associated with being a SMA could influence adolescent neurodevelopment and affective states. Finally, while images of all genders were included in the individualized paradigms, we did not control for attraction and it is likely that all participants felt some degree of romantic/sexual attraction to some of the peer images irrespective of sexual orientation. While there is evidence for neural correlates of sexual reward and attraction (Gola et al., 2015; Eckstrand et al., 2017), whether there are detectable neural differences between SMA and heterosexual adolescents remains unclear. The degree to which sexual/romantic attraction is influencing the presented results is unknown and is a potential area for future research.

Even with the apparent relevance of these findings to affective psychopathology in SMA, the present study is clearly preliminary given the small sample size and results should be interpreted with caution. Given the small size of the SMA group, there is the chance for Type II error or the chance that we were underpowered to detect smaller but meaningful group differences. Several methods were applied to support the replicability and power of the data. First, covariates that potentially influence the presented results—including age, gender, and race—were corrected for in the imaging model. Second, a multiple linear regression was performed to minimize multiple comparisons. All models were performed with bootstrapping, supporting the reliability of findings and stability of the data. Lastly, *post hoc* power analyses supported that the presented data were adequately powered to detect medium-to-large effects (see Supplementary Figure S2). While these tests better characterize the clear limitations of the sample size and results, larger studies drawn from a population with a wider range of clinical depressive symptoms—with ample power to detect smaller group differences and sample sizes robust to the influence of outliers—will be critical for elucidating the presence and meaning of differences (Poldrack et al., 2017). However, based on the striking disparities in interpersonal stress-related depression and suicide risk among SMA, it is critical to further explore the relationships between interpersonal interactions related to sexual identity development and the neural circuits underlying the development of depression. Such research is important, even in those who are psychiatrically healthy, particularly given that adolescents normatively have higher depressive symptoms and that examining subthreshold clinical variability may be helpful in understanding individual risk.

Despite these limitations, the present study is noteworthy for being the first to examine differences in neural activation between SMA and heterosexual adolescents. It offers novel initial findings suggesting that blunted neural responses to social, but not monetary, reward in socioaffective processing regions distinguish SMA from heterosexual adolescents and may serve as a mechanism for depression. These findings overlap with the observed disparity in—and provide a plausible neural signature for the development of—depression prevalence among SMA. Further, these findings can provide important guidance for future studies using prospective designs and sampling from broader SMA populations to explore the interaction between social experiences, brain development, to potentially lead to depression among SMA.

## Data Availability

The datasets generated for this study are available on request to the corresponding author.

## Ethics Statement

This study was carried out in accordance with the recommendations of the University of Pittsburgh Institutional Review Board with written informed consent from all subjects. All subjects gave written informed consent in accordance with the Declaration of Helsinki. The protocol was approved by the University of Pittsburgh Institutional Review Board.

## Author Contributions

KE, JS, NA, MM and EF contributed to the conception of the work. KE, LF, MC and KH contributed to the acquisition, analysis, and interpretation of data for the work. KE, LF, MM and EF drafted and revised the work for important intellectual content. All authors gave final approval for publication of the work and agree to accountable for the accuracy and integrity of the work.

## Conflict of Interest Statement

The authors declare that the research was conducted in the absence of any commercial or financial relationships that could be construed as a potential conflict of interest.

## Footnotes

^1^http://www.fil.ion.ucl.ac.uk/spm

^2^https://www.nitrc.org/projects/artifact_detect

^3^http://restfmri.net/forum/REST_V1.8

^4^http://marsbar.sourceforge.net/
