# Supplementary material for: Social and Non-social Reward Processing and Depressive Symptoms Among Sexual Minority Adolescents
Source: Front Behav Neurosci. 2019 Sep 13;13:209. doi: 10.3389/fnbeh.2019.00209 (PMC6753189; doi:10.3389/fnbeh.2019.00209)

**Supplementary Material**

**S1. Methods**

**S.1.1. Victimization.** Items assessing victimization in the composite measure were: (*a*) “During the past 12 months, how many times were you in a physical fight on school property?” (*b*) “During the past 12 months, how many times were you in a physical fight?” (*c*) “During the past 12 months, how many times has someone threatened or injured you with a weapon such as a gun, knife, or club on school property?” (*d*) “During the past 12 months, how many times were you in a physical fight in which you were injured and had to be treated by a doctor or nurse?” (*e*) “During the past 30 days, on how many days did you carry a weapon such as a gun, knife, or club?” (*f*) “During the past 30 days, on how many days did you carry a gun?” (*g*) “During the past 30 days, on how many days did you carry a weapon such as a gun, knife, or club on school property?” Response options for questions *a-c* were zero times [coded as 0], 1 time [1], 2 or 3 times [2], 4 or 5 times [3], 6 or 7 times [4], 8 or 9 times [5], 10 or 11 times [6], and 12 or more times [7]. Response options for questions *d-g* were zero times [coded as 0], 1 time [1], 2 or 3 times [2], 4 or 5 times [3], and 6 or more times [4]. Two additional questions addressing bullying and safety were included: (*h*) “During the past 30 days, on how many days did you not go to school because you felt you would be unsafe at school or on your way to or from school?” with response options of zero days [coded as 0], 1 day [1], 2 or 3 days [2], 4 or 5 days [3], and 6 or more days [4], and (*i*) “Have you been bullied on school property?” with response options of no [0] or yes [1].

**S.1.2. Power and reliability measurement.** There are two sets of analyses in the current study: determining whether there are differences in neural activation in response to social reward between sexual minority adolescents (SMA) and their heterosexual peers, and whether differences in activation are associated with depressive symptoms. Recent research has addressed the issue of power and robustness in smaller samples in both neuroimaging (Button et al., 2013; Eklund, Nichols, & Knutsson, 2016) and statistical models (Green and MacLeod, 2016). The power of the neuroimaging analysis based on the task type, sample size, smoothing kernel and statistical methods is discussed in the Results.

Two analyses were conducted to test the power and robustness of the multiple linear regression model examining associations between depressive symptoms and neural activation differences to social reward based on sexual orientation. The first, completed in G*Power (Faul, Erdfelder, Lang, & Buchner, 2007), was a post hoc power analysis where the effect size of the predictors in the statistical model (calculated as η^2^), sample size, and α (0.05) were used to calculate the achieved power. Based on these parameters, power was interpolated based on a function of varying sample and effect sizes. The second utilized bootstrapping in SPSS to demonstrate the reproducibility of the second aim of the study. The bootstrap analysis was performed with 10,000 resamples in the multiple linear regression model examining the association between neural activation differences based on sexual orientation and depressive symptoms.

**S2. Results**

**S2.1. Power of imaging model**

Recent concern has been raised about the use of less stringent cluster determining thresholds (CDT) in neuroimaging analyses leading to high false positive rates (Eklund, Nichols, & Knutsson, 2016; Woo, Krishnan, & Wager, 2014). The seminal paper by Eklund et al. examines the false positive rate in three independent samples (with sample sizes of 20-40 subjects) across image analysis platforms using four different functional MRI tasks, finding that the false positive rate is affected most by the CDT, smoothing kernel, and task type. Specifically, less stringent CDTs, smaller smoothing kernels, and event-related designs had a higher false positive rate. In two-sample tests with a longer-block block design, Eklund et al. found that false positive rates ranged between 10-20% with a CDT of *p*<0.01 and 3-7% with a CDT of *p*<0.001 (see Eklund, et al., 2016, supplementary figures 3-4, 7-8). Importantly, permutation testing negated the negative effects of less stringent CDTs, smoothing kernel, and task, typically yielding false positive rates of less than 5% (see Eklund, et al., 2016, supplementary table 1).

In the present study, we utilized SPM and a 6mm smoothing kernel to analyze data from a social reward task with block design composed of longer blocks. At the recommended voxel-wise inference level of *p*<0.001, three main regions – each composed of 2-3 non-contiguous clusters – emerged as being less active among SMA (see Supplementary Table 3). The same three regions remained active using a CDT of *p*<0.005 in combination with permutation testing (i.e. Monte Carlo simulations); however, the clusters were contiguous using this approach (see Table 2). The peak voxel in each of these clusters remains significant at *p*<0.001, as demonstrated above, and both approaches would be expected to yield a false positive rate of ≤5% based on the analyses performed by Eklund et al. As such, the presented neuroimaging findings do meet accepted statistical thresholds.

**S2.3. Power of Multiple Linear Regression Model**

In the multiple linear regression model, the calculated effect size of association between right TPJ activation and depressive symptoms is *f*^2^=0.27 and calculated power is 0.80 (see Supplementary Figure 2). Supplementary Figure 2 presents the power of the multiple linear regression model as a function of sample size with typical large (β = 0.35), medium (β = 0.15), and small (β = 0.02) effects. Each line represents the proportion of samples wherein the effect was successfully detected at p < .05. The grey region represents sample sizes that exceed the conventional standard for power (i.e., > 0.80), and the vertical red line represents the final sample size for report results (N = 45). As can be seen, this sample size is sufficiently powered with a power of 0.80 (power to detect the effect size observed in this study [β = .27, blue line]), suggesting that our current sample has power for detecting medium-to-large effects. As included in the discussion, this supports the assertion that while we are powered to detect larger effects, we are underpowered to detect smaller but meaningful differences between groups.

Further demonstrating the replicability of the multiple linear regression model, the critical association between right TPJ activation and interpersonal depressive symptoms remains significant after bootstrap methods (β=-0.82, p=0.05, 95% CI[-1.69,-0.18], see Supplementary Table 4). It is also worth noting that none of the significant associations from the multiple linear regression lost significance in the bootstrap model, further suggesting the stability of the model. Combined, these two additional tests suggest that the study is adequately powered to test the second aim – whether neural activation differences based on sexual orientation are associated with depressive symptoms.

**References**

Button, K. S., Ioannidis, J. P., Mokrysz, C., Nosek, B. A., Flint, J., Robinson, E. S., & Munafo, M. R. (2013). Power failure: why small sample size undermines the reliability of neuroscience. *Nature reviews neuroscience, 14*(5), pp. 365-376.

Eklund, A., Nichols, T. E., & Knutsson, H. (2016). Cluster failure: Why fMRI inferences for spatial extent have inflated false-positive rates. *Proceedings of the national academy of sciences of the USA, 113*(28), pp. 7900-7905.

Faul, F., Erdfelder, E., Lang, A. G., & Buchner, A. (2007). G*Power 3: a flexible statistical power analysis program for the social, behavioral, and biomedical sciences. *Behavioral research methods, 39*(2), pp. 175-191.

Green, P., & MacLeod, C. J. (2016). SIMR: an R package for power analysis of generalized linear mixed models by simulation. *Methods in ecology and evolution, 7*(4), pp. 493-498.

Woo, C.-W., Krishnan, A., & Wager, T. D. (2014). Cluster-extent based thresholding in fMRI analyses: pitfalls and recommendations. *Neuroimage, 91*, pp. 412-419.

| **Supplementary Table 1.** Victimization Experiences Among Adolescents | |  | |
| --- | --- | --- | --- |
|  | | **Orientation** | |
|  |  | **Straight** | **SMA** |
|  |  | N | N |
| Skipped School Because You Felt Unsafe | 0 days | 36 | 8 |
| Bullied on School Property | No | 35 | 8 |
|  | Yes | 1 | 0 |
| Carried a Weapon | 0 days | 34 | 7 |
|  | 1 day | 1 | 0 |
|  | 2 or 3 days | 1 | 1 |
| Carried a Gun | 0 days | 36 | 8 |
| Carried a Weapon on School Property | 0 days | 36 | 7 |
|  | 1 day | 0 | 0 |
|  | 2 or 3 days | 0 | 1 |
| Threatened With Weapon on School Property | 0 times | 36 | 8 |
| Been in a Fight Where You Went To The Hospital or Doctor | 0 times | 36 | 8 |
| Been in a Fight on School Property | 0 times | 34 | 7 |
|  | 1 time | 2 | 0 |
|  | 2 or 3 times | 0 | 1 |
| Been in a Fight | 0 times | 31 | 7 |
|  | 1 time | 1 | 0 |
|  | 2 or 3 times | 5 | 1 |

| **Supplementary Table 2**. Depressive Symptoms and Victimization by Sexual Orientation without Outlier Winsorization | | | | | |  |
| --- | --- | --- | --- | --- | --- | --- |
|  | | *Heterosexual* | *SMA* |  | *p* | |
|  |  | Mean±SD | Mean±SD | Z-score |  |  |
| CES-D^†^ | Somatic | 4.00±3.62 | 2.38±2.20 | -1.04 | 0.30 | |
|  | Depressive | 2.16±3.15 | 2.88±2.23 | -1.19 | 0.27 | |
|  | Positive Affect | 2.63±2.79 | 2.13±2.03 | -0.28 | 0.79 | |
|  | Interpersonal | 0.47±1.18 | 1.13±1.36 | -2.16 | **0.03** | |
| Victimization^†^ |  | 0.09±0.24 | 0.20±0.37 | -0.48 | 0.69 | |

CES-D, Center for Epidemiologic Studies Depression Scale.
^†^ Performed with bootstrapping (n=10,000 resamples)
Bold values indicate *p*<0.05.

**Supplementary Table 3.** Bootstrapped multiple linear regression model with and without demographic covariates

|  |  |  |  |  |  |  |  |  |  |
| --- | --- | --- | --- | --- | --- | --- | --- | --- | --- |
| **Without Covariates** | | | | |  | **With Covariates** | | | |
| CESD Subscale | | β | *p* | 95% Confidence Interval | | β | *p* | 95% Confidence Interval | |
|  |  |  |  | LLCI | ULCI |  |  | LLCI | ULCI |
| Somatic | RTPJ | -1.95 | 0.02 | -3.99 | -0.45 | -2.24 | 0.04 | -4.58 | -0.49 |
|  | Lins | 4.63 | 0.01 | 1.52 | 8.35 | 5.12 | 0.01 | 1.62 | 9.57 |
|  | mPFC | 0.50 | 0.47 | -1.05 | 1.90 | 0.37 | 0.63 | -1.52 | 1.91 |
| Depressive | RTPJ | -1.07 | 0.19 | -2.91 | 0.57 | -1.31 | 0.17 | -3.30 | 0.53 |
|  | Lins | 0.88 | 0.60 | -2.47 | 4.21 | 1.17 | 0.46 | -2.24 | 4.33 |
|  | mPFC | 0.88 | 0.25 | -0.61 | 2.46 | 0.74 | 0.32 | -0.64 | 0.90 |
| Positive | RTPJ | 0.36 | 0.64 | -1.38 | 1.99 | 0.10 | 0.91 | -1.16 | 1.88 |
|  | Lins | 0.36 | 0.88 | -4.84 | 4.52 | 1.01 | 0.75 | -5.20 | 5.91 |
|  | mPFC | 0.27 | 0.70 | -0.98 | 2.09 | 0.12 | 0.88 | -1.32 | 2.13 |
| Interpersonal | RTPJ | -0.82 | 0.05 | -1.69 | -0.18 | -0.89 | 0.05 | -1.81 | -0.17 |
|  | Lins | 0.92 | 0.07 | -0.06 | 1.90 | 1.00 | 0.08 | -0.17 | 2.10 |
|  | mPFC | <-0.01 | 0.99 | -0.45 | 0.59 | -0.03 | 0.91 | -0.53 | 0.69 |

LIns, left insula; mPFC, medial prefrontal cortex; RTPJ, right temporoparietal junction

**Supplementary Figure 1.** Data outliers before and after winsorization.


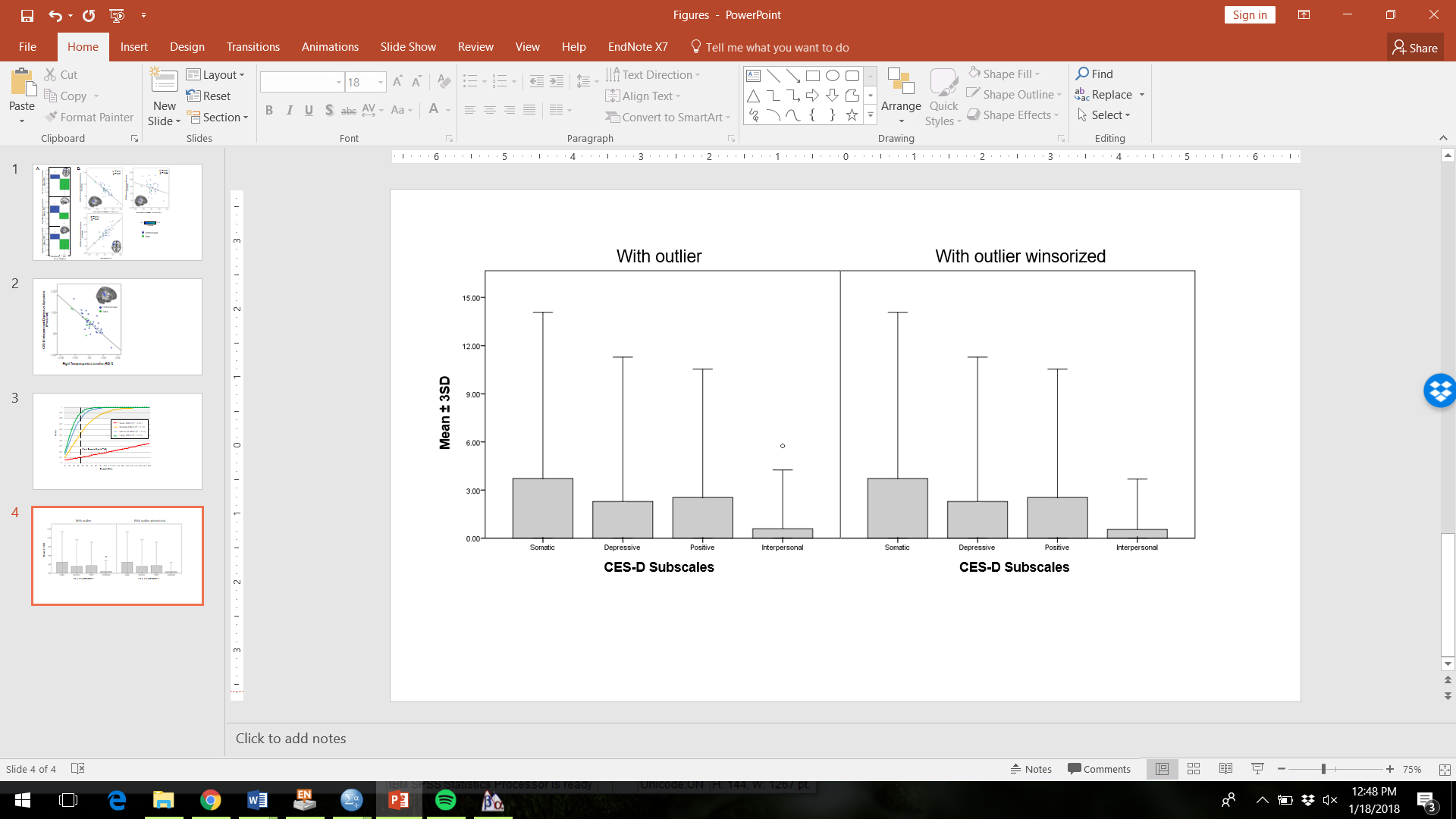


**Supplementary Figure 2.** Simulated power analyses demonstrating that current study is powered to detect medium-to-large effects.


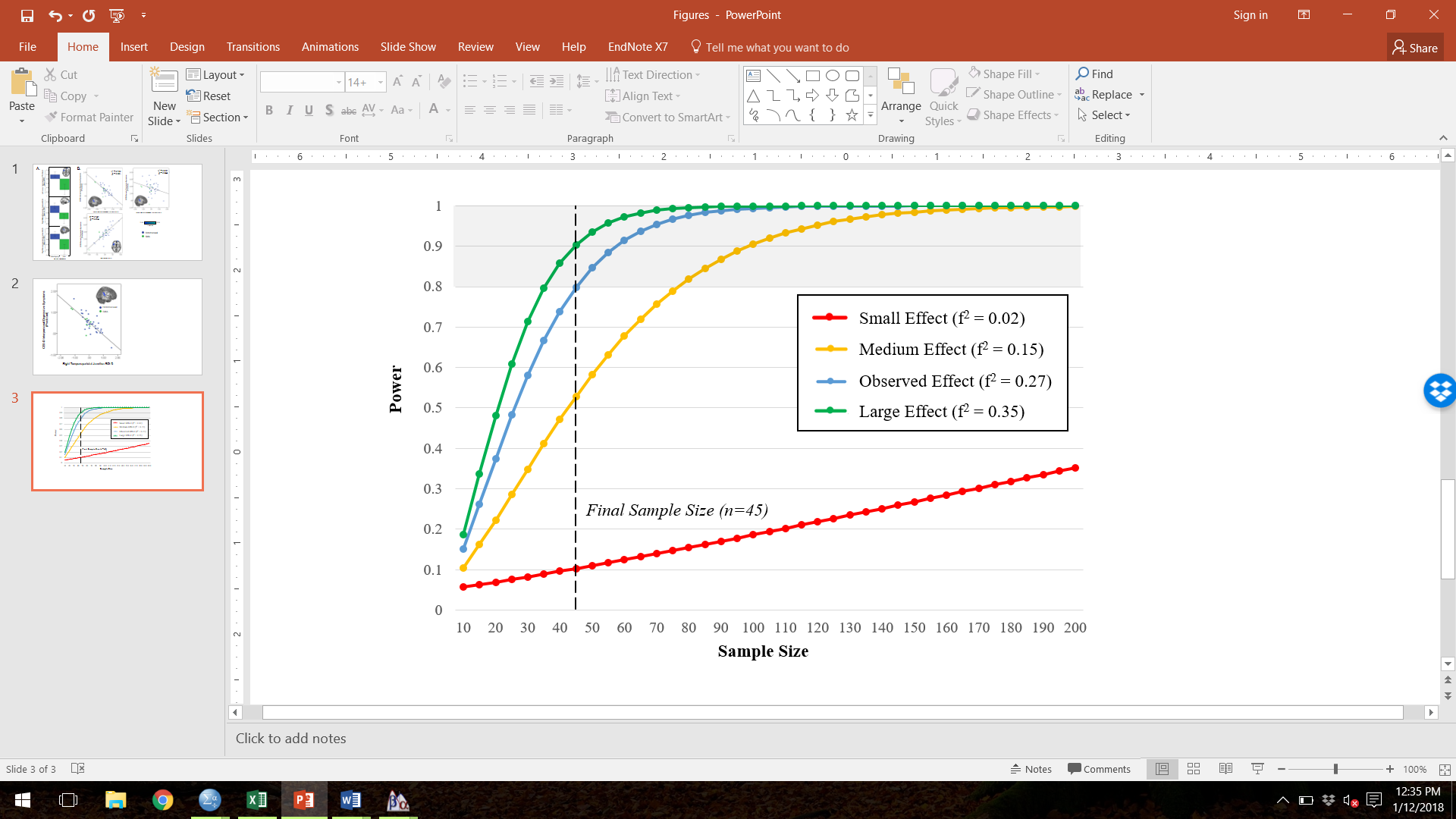

Supplement: Supplementary file 1 [file Data_Sheet_1.docx]
